# Taurolidine lock solution for catheter-related bloodstream infections in pediatric patients: A meta-analysis

**DOI:** 10.1371/journal.pone.0231110

**Published:** 2020-04-07

**Authors:** Yan Sun, Guanghui Wan, Liping Liang

**Affiliations:** Zaozhuang Traditional Chinese Medicine Hospital, Zaozhuang, Shandong, P.R. China; University Magna Graecia of Catanzaro, ITALY

## Abstract

Infection is one of the most commonly described complications, and a major cause of morbidity and mortality in pediatric patients treated using central venous catheters (CVCs). Taurolidine lock solutions have been used to decrease catheter-related bloodstream infections (CRBSIs) in both adult and pediatric patients. The purpose of this study was to systematically search the literature and conduct a meta-analysis to determine the efficacy of taurolidine in reducing CRBSI in children. We conducted an electronic search of the PubMed, EMBASE, Cochrane Library, TRIP Database, CINAHL, and Google Scholar databases for articles published up to 1^st^ November 2019. Eligible studies included randomized controlled trials (RCTs) comparing the effects of taurolidine with control for preventing CRBSI in pediatric patients. Four studies were included. Our results indicated a statistical significant reduction in the total number of CRBSI with taurolidine as compared to control (RR: 0.23; 95% CI:0.13, 0.40; I^2^ = 0%; P<0.00001). The pooled analysis also indicated a statistical significant reduction in the incidence of CRBSI (defined as the number of CRBSI events/1000 catheter days) in the taurolidine group (MD: -1.12; 95% CI:-1.54, -0.71; I^2^ = 1%; P<0.00001). The number of catheters removed due to infection or suspected infection was not significantly different between the two groups (RR: 0.68; 95% CI:0.22, 2.10; I^2^ = 56%; P = 0.50) (Fig 5). The quality of the included studies was not high. The use of taurolidine as a catheter locking solution may significantly reduce CRBSI in pediatric patients. However, the quality of current evidence is not high and further high-quality large scale RCTs are needed to corroborate our results.

## Introduction

Central venous catheters (CVCs) are often an inevitable part of treating adult and pediatric patients receiving antibiotic therapy, parenteral nutrition, hemodialysis, chemotherapy, or hospitalized in Intensive Care Units. (ICU) [1]. However, the long-term use of CVCs carries a risk of catheter-related bloodstream infections (CRBSI) that are associated with increased morbidity and mortality rates. According to the Centre for Disease Control and Prevention, an estimated 41,000 new cases of CRBSI are diagnosed annually and the disease has a mortality rate of 12–15% in the general population [2]. Prevention of CRBSI is therefore pivotal to improve clinical outcomes in high-risk patients.

Intraluminal contamination, especially with Staphylococci, is considered the main cause for CRBSI, and the risk of infection further increases with long-term catheter use [3]. The most frequent mechanism of CRBSI infection is catheter hub contamination during catheter manipulation by healthcare professionals leading to endoluminal colonization and infection. CRBSI rates are high in pediatric patients (over 10 per 1000 catheter-days) and are associated with increased mortality and treatment costs [4,5].

Heparin which is routinely used to lock the catheter to prevent clotting is thought to be associated with the growth of bacteria inside the catheter hub [6]. While prophylactic antimicrobial lock solutions are beneficial in preventing CRBSI, they also increase the risk of selecting resistant microorganisms [7]. Taurolidine [bis-(1,1-dioxoperhydro-1,2,4-thiadiazinyl-4)-methane], a derivative of the amino acid taurine, is an antimicrobial agent with a broad range of antibacterial and antifungal activity that includes both Gram-positive and Gram-negative bacteria, such as methicillin-resistant Staphylococcus aureus (MRSE), coagulase-negative Staphylococci (CoNS), and vancomycin-resistant enterococci [8]. Taurolidine has few reported side-effects, and its continued use is not associated with developing bacterial resistance [9]. Both retrospective, as well as prospective studies, have reported a reduction in the number of CRBSIs after the use of taurolidine lock in the catheter lumen [10–12].

In a systematic review and meta-analysis of six randomized controlled trials (RCTs) published in 2013, Liu et al [13] have reported a 66% reduced risk of CRBSI when taurolidine is used as a locking solution. Their review, however, included a mix of four adult and two pediatric studies. In a recent systematic review of 2017, Norris et al [13] have discussed the effectiveness and safety of prophylactic antimicrobial lock solutions in adults as well as pediatric cancer patients with CVCs. Their review was, however, not focused specifically on taurolidine and was a literature summary of all types of studies evaluating all types of antimicrobial lock solutions. To the best of our knowledge, to date, no study has pooled evidence to analyze the effectiveness of taurolidine lock in reducing CRBSI in pediatric patients. Therefore, the purpose of this study was to systematically search the literature and perform a meta-analysis to determine the efficacy of taurolidine in reducing CRBSI in pediatric patients.

## Methods

### Study design and literature search

This review was conducted following the guidelines of the PRISMA statement (Preferred Reporting Items for Systematic Reviews and Meta-analyses) [14] and the Cochrane Handbook for Systematic Reviews of Intervention [15]. We conducted an electronic search of the PubMed, EMBASE, Cochrane Library, TRIP Database, CINAHL and Google Scholar databases using the MeSH or free text terms for articles published up to 1^st^ November 2019. Google Scholar was searched for only the first 200 results for each search query. The search was restricted to studies performed on human pediatric patients (<19 years of age). No restriction on language or publication period was set. Reference lists of all included studies, as well as review articles on the subject, were manually inspected for additional relevant articles.

Search terms included “central venous catheters”, “infection”, “catheter-related infection", "antimicrobial lock solution", "antibiotic lock solution", “taurolidine”, “taurolidine citrate”, “heparin”, “pediatrics”, “prevention”, and “children”. Detailed search strategy and results of the PubMed database are presented in S1 Table. Two reviewers (Y.S. & G.W.) conducted the search independently. Any disagreement with regards to inclusion/exclusion of studies were resolved by discussion with the third reviewer (L.L.).

### Selection criteria

We used the PICOS (Population, Intervention, Comparison, Outcome, and Study design) model to select studies for this review. Only RCTs conducted on pediatric patients (<19 years of age) implanted with a CVC for any reason *(Population*), were included. Studies were to compare taurolidine lock solution *(Intervention)* with control (heparin or saline or no lock) *(Comparator)* for the reduction of CRBSI *(Outcome)*. There was no restriction on the concentration of taurolidine or heparin used by the trials. Also, no restrictions were placed regarding language or sample size. In studies wherein both adult and pediatric patients were analyzed, the trial was included only if pediatric data was separately retrievable. Studies on adult patients, studies utilizing any other antimicrobial or alcohol lock solutions, single-arm studies, non-RCTs, abstracts, duplicates, case reports and case studies were excluded.

### Data collection and analysis

Data were extracted independently by two reviewers using a pre-designed form that included the first author’s name, year of publication, study design, country of research, demographic details, sample size, catheter duration, locking protocol, use of prophylactic antibiotics, patients lost to follow-up, study outcomes, and complications. Any disagreement was resolved by discussion with the third reviewer. Missing data were requested from the original authors through electronic correspondence or mail.

The primary outcome of interest was the total number of CRBSI while secondary outcomes were incidence rate of CRBSI (defined as the number of CRBSI events/1000 catheter days) and the number of CVC removed due to infection or suspected infection. Other outcomes studied but not included in the meta-analysis involved time interval from the start of locking and infection and incidence of catheter thrombosis. Definitions of CRBSI was as per the included study.

### Risk of bias in individual studies

The quality of included studies was assessed using the Cochrane Collaboration risk assessment tool for RCTs [15]. Risk of bias (low, unclear or high) was gauged for random sequence generation, allocation concealment, blinding of participants and personnel, blinding of outcome assessment, incomplete outcome data, selective reporting, and other biases.

### Statistical analysis

Data were analyzed on an intention-to-treat basis. Continuous data were pooled using Mean Difference (MD) and 95% confidence interval (CI). Categorical data were summarized using the Mantel-Haenszel Risk Ratio (RR) and 95% CI. The I^2^ statistic was used to assess heterogeneity wherein values of 25–50% denoted low, 50–75% denoted medium and values of more than 75% denoted considerable heterogeneity. Considering the heterogeneity in the patient sample, the number of catheter days and other methodological variations amongst the included studies, a random-effects model was preferred for the meta-analysis. Review Manager (RevMan, version 5.3; Nordic Cochrane Centre [Cochrane Collaboration], Copenhagen, Denmark; 2014) was used for the meta-analysis. In studies reporting only 95% CI for continuous variables, standard deviations were calculated using the in-build RevMan calculator. A sensitivity analysis was carried out to assess the influence of each study on the pooled effect size. Using the one-study-out method, we evaluated whether deleting each individually would significantly change the results of the meta-analysis. Due to the limited number of included studies, publication biased was not assessed.

## Results

### Identification of relevant studies

**Fig 1:**
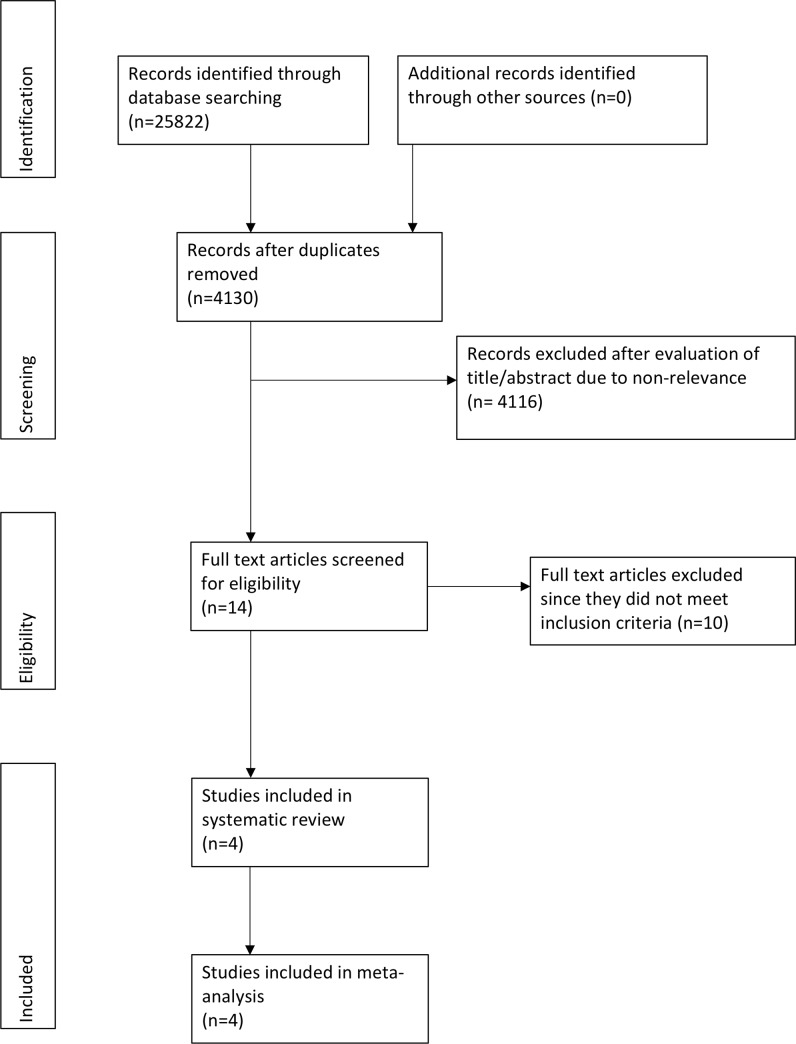
Flow diagram of the selection of studies and specific reasons for exclusion from the present meta-analysis.

The study flow chart is presented in Fig 1. Eleven articles were selected for full-text analysis. Ten studies were excluded as two were retrospective studies [16,17], one was a non-RCT [18], one was a single-arm trial [19] while six studies were carried out on adult patients [20–25]. A total of four studies [26–29] were included in this systematic review and meta-analysis.

All of the included studies followed the Declaration of Helsinki for the ethical treatment of humans in experimentation which was developed by the World Health Organization. Table 1 summarizes the characteristics of the selected studies.

**Table 1:** Characteristics of the published studies included in meta-analysis.

Author & Year	Country	Patient population	Setting/ centers	Sample size		Age in years (Median) range		Male gender		Cumulative Catheter Duration (days)		Time of taurolidine lock	Control group protocol	Prophylactic Antibiotics used	Patients lost to follow up	
				Taurolidine	Control	Taurolidine	Control	Taurolidine	Control	Taurolidine	Control				Taurolidine	Control
Simon *et al*. 2008 [27]	Switzerland	Cancer	Tertiary care hospital	89	90	7.2 (3.7–16.1)	10.4 (5.2–14.7)	60	51	6705	6086	Once or twice a week	200 IU heparin lock in 2ml of normal saline	Cotrimoxazole used for prevention of Pneumocystis jirovecii pneumonia	NS	NS
Dumichen *et al*. 2012 [26]	Germany	Neoplastic Disease	Tertiary care hospital	35	36	7.5 (1.4–18.0)	6.3 (1.7–17.1)	19	23	6576	7233	NS	100 IU heparin/ml of normal saline	Cotrimoxazole used for prevention of Pneumocystis jirovecii pneumonia	10	10
Handrup *et al*. 2013 [29]	Denmark	Cancer	Tertiary care hospital	64	65	6 (0–19)	5 (0–16)	45	33	39127*		After each treatment cycle	250 IU of heparin in 2.5 ml of normal saline	NS	0	0
Lyszkowaska *et al*. 2019 [28]	Poland	Surgical treatment with observation	Tertiary care hospital	48	49	<2	<2	NS	NS	942	946	Between intervals of parenteral nutrition or intra-venous drug supply	No locking solution	Standard perioperative prophylaxis or for treatment of co-existing bacterial infection	0	0

*Combined data of study and control groups

NS, Not specified

All studies were RCTs carried out in European countries in a tertiary care setting. The study population consisted of cancer patients in three trials [26,27,29] while one study included patients requiring CVC during and after surgical procedures (for parenteral nutrition or drug administration) [28]. The sample of included studies ranged from 35–90 patients per group. There was a wide variation in the cumulative duration of catheter use in the included studies. The concentration of taurolidine was 1.35% with 4% sodium citrate in all studies. Three studies [26,27,29] used heparin locks in the control group while no locking solution was used in the control group of one trial [28]. Three trials used prophylactic antibiotics during the course of the study [26–28]. Duration of locking was specified in only one study wherein the mean duration was 172.6 in the taurolidine group and 187.4 days in the control group [26].

**Fig 2:**
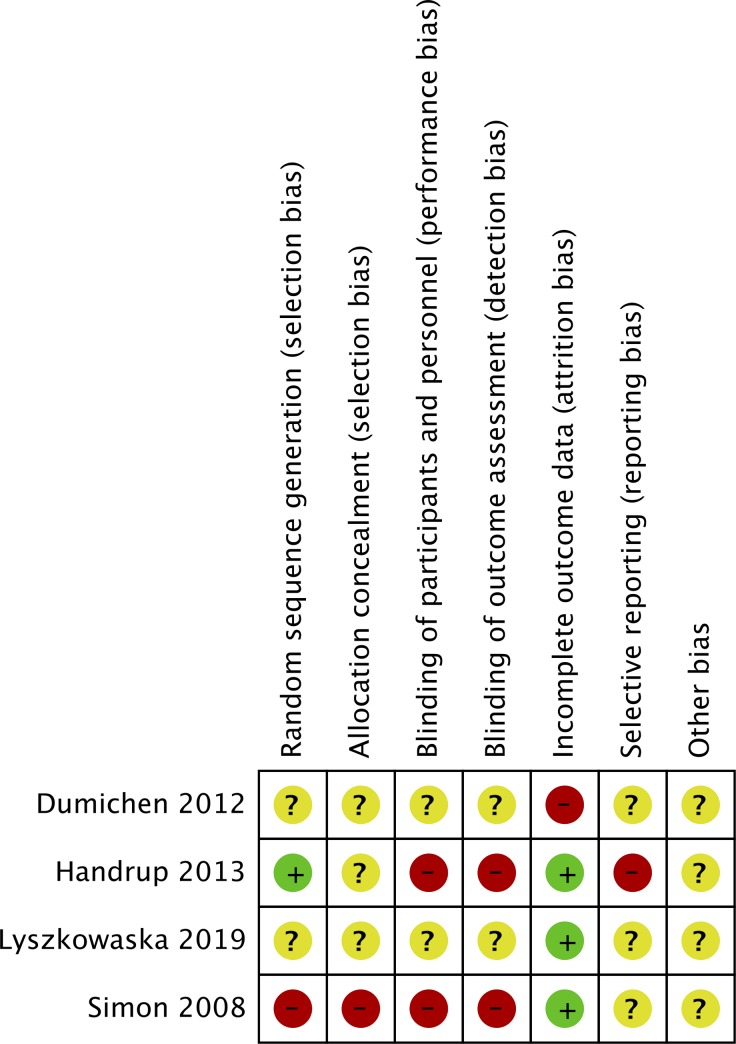
Risk of bias summary of included studies. Red circle denotes a high risk of bias, Yellow circle denotes an unclear risk of bias and green circle denotes a low risk of bias.

The authors' judgment of the risk of bias in the included studies is presented in Fig 2. Adequate method of randomization was utilized in only one trial [29]. None of the trials were blinded. Attrition bias was significant in one study [26]. None of the trials were pre-registered.

The definition of CRBSI in the included studies and summary of outcomes is presented in Table 2. CRBSI was studied by all four included trials. Time interval from the start of locking and first infection was reported by two trials [26,29] with no significant difference between the two groups. All included studies also did not report any significant difference between the incidence of catheter thrombosis between the two groups.

**Table 2:** Definition and outcomes in included studies.

Author & Year	Definition of catheter related CRBSI	Total number of CRBSI		Time interval from start of locking and infection (Mean ± SD)		Incidence rate of BSI* (95% CI or SD)		Number of catheter removal due to infection		Catheter thrombosis	
		Taurolidine	Control	Taurolidine	Control	Taurolidine	Control	Taurolidine	Control	Taurolidine	Control
Simon *et al*. 2008 [27]	Clinical signs of infection plus at least two positive blood cultures for coagulase-negative staphylococci (CoNS) or methicillin resistant staphylococci (MRSE) taken from a CVC, and no evidence of another primary focus of infection	3	14	NS	NS	0.45 (0.09–1.31)	2.30 (1.26–3.86)	3	4	0	0
Dumichen *et al*. 2012 [26]	Patient with CVC has a recognized pathogen cultured from one or more blood cultures, and the organism cultured from blood is not related to an infection at another site	2	9	35.6± 31.8	41.2± 49.4	0.3 (1.2)	1.3 (2.5)	5	2	3	2
Handrup *et al*. 2013 [29]	Patient with CVC has a recognized pathogen cultured from one or more blood cultures; or a common skin contaminant cultured from two or more blood cultures, both drawn at separate occasions. In both cases, the cultured organism must not be related to pathogens identified at other infection sites	7	26	300 ± NR	156 ± NR	0.4 (0.17–0.78)	1.4 (0.93–2.01)	5	7	0	0
Lyszkowaska *et al*. 2019 [28]	Deterioration of the patient’s condition, an increase or decrease in the number of white blood cells, thrombocytopenia, anaemia, positive blood culture and exclusion of other sources of infection.	1	14	NS	NS	1.06 (0.096–4.96)	14.3 (8.18–23.35)	1	11	1	1

CVC, Central venous catheter; CRBSI, catheter-related bloodstream infection; SD, standard deviation; NR, Not reported; NS, Not studied; CI, Confidence intervals

### Meta-analysis

**Fig 3:**
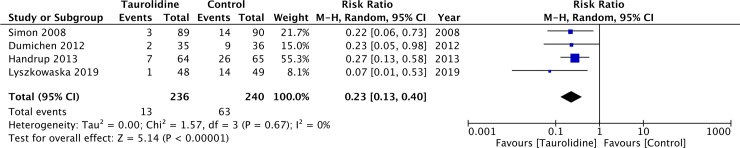
Forest plot of taurolidine versus control for the total number of CRBSI.

**Fig 4:**
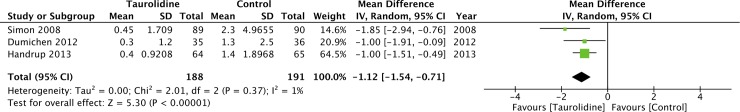
Forest plot of taurolidine versus control for an incidence rate of CRBSI.

**Fig 5:**
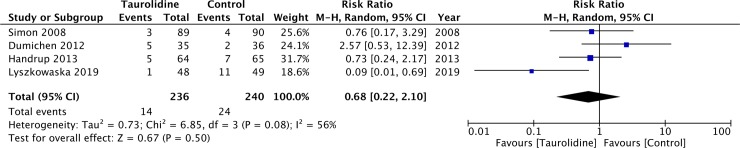
Forest plot of taurolidine versus control for the number of catheters removed due to infection.

For the primary outcome of the total number of CRBSI, data of 236 patients in the taurolidine group was compared with data of 240 controls. Our results indicated a statistical significant reduction in the total number of CRBSI with taurolidine as compared to control (RR: 0.23; 95% CI:0.13, 0.40; I^2^ = 0%; P<0.00001) (Fig 3). Data on incidence rates of CRBSI was available from three studies [26,27,29]. Pooled analysis indicated a statistical significant reduction in the incidence of CRBSI in the taurolidine group (MD: -1.12; 95% CI:-1.54, -0.71; I^2^ = 1%; P<0.00001) (Fig 4). The number of catheters removed due to infection or suspected infection was not significantly different between the two groups (RR: 0.68; 95% CI:0.22, 2.10; I^2^ = 56%; P = 0.50) (Fig 5). On sensitivity analysis, there was no change in the significance of the results of both primary and secondary outcomes on the exclusion of any study.

## Discussion

According to the Centers for Disease Control (CDC), steps for the prevention of CRBSI should include handwashing, use of aseptic technique, site cleansing and use of impregnated catheter cuffs [2]. Regardless of these precautions, CRBSI continues to occur leading to a prolonged hospital stay, higher health-care cost as well as increased morbidity and mortality. Over the past decade, several antimicrobial catheter locking solutions have been used to reduce the incidence of CRBSI but with varying results [30]. The primary aim of this systematic review and meta-analysis was to analyze if the use of one such locking solution, i.e. taurolidine, reduces the risk of CRBSI in pediatric patients. The results of our study indicate that taurolidine may significantly reduce the total events of CRBSI as well as the incidence rate of CRBSI in the pediatric population.

The long term use of CVCs has been associated with intraluminal contamination and subsequent infection [31]. A microbial biofilm is formed in the catheter lumen from contamination either from the infusate or catheter hub, detachment of which, may lead to CRBSI [32]. Children are particularly high-risk candidates for CRBSI with incidence rates exceeding 10 per 1000 catheter days [4,5]. As biofilms are resistant to routine systemic antimicrobial therapy, the use of antimicrobial locking solutions may be beneficial in reducing the incidence of CRBSI. The advantage of the antimicrobial locking solution is that it maintains a high concentration of the agent in close contact with the catheter lumen for a prolonged period, with minimal systemic absorption [33]. Studies have reported that taurolidine is effective in reducing the microbial load of biofilms in hemodialysis patients requiring long-term catheterization [32]. A number of RCTs conducted on adult patients requiring long-term catheterization for hemodialysis [24], parenteral support [22] or cancer chemotherapy [20], have also demonstrated a reduced risk of CRBSI with the use of taurolidine lock solution. The outcomes of our analysis concur with the result of these trials.

In our review, the total number of CRBSI events were 13/236 (5.5%) in the taurolidine group and 63/240 (26.25%) in the control group, with a statistically significant 77% reduced risk of CRBSI in the taurolidine group. To account for the difference in duration of catheter use amongst the included trials, we also analyzed the incidence rate of CRBSI between the two groups. Our results indicate a statistical significant reduction of CRBSI incidence/1000 catheter days with the use of taurolidine. The results are similar to the previous meta-analysis of the taurolidine lock solution by Liu *et al*[13], who reported a 66% reduced risk of CRBSI. Their study was however not focused on pediatric patients. As a secondary outcome, we also analyzed the total number of catheters removed due to infection or suspected infection. Our analysis indicated no significant difference between the two groups. The non-significant results can be explained by the differences in catheter removal criteria as well as the severity and management protocol of CRBSI in the included trials. Also, CVCs were removed even in cases of suspected infection in one study. But, the subsequent culture of the catheter did not demonstrate bacterial growth in all cases [26].

On analysis of the results of individual studies, Dumichen *et al* [26] was the only study to report a minimal difference in the total number of CRBSI events (RR: 0.23, 95% CI: 0.05–0.98) as well as incidence rates of CRBSI between the two groups. This may partly be explained by the limited sample size of the study and the high attrition rate of the trial. The authors had recognized that their study was underpowered to detect statistical significant differences between the two groups.

Several factors can influence the risk of CRBSI in patients with CVCs. These include the patient's medical history, duration of catheterization, frequency and duration of locking solutions, use of other antiseptic measures, prophylactic antibiotics, etc. It is assumed that the role of such confounding factors is negated by conducting a robust RCT in a single center to maintain homogeneity of the protocol. However, when a meta-analysis combines the results of multiple RCTs, inter-study heterogeneity can influence the study results. In the four RCTs combined in our analysis, one study assessed the efficacy of taurolidine lock solutions only in surgical patients. Data on the frequency and total duration of locking were not provided by all included trials. Also, there were differences in the cumulative duration of catheterization in the included studies. While the total duration of catheterization was less than 1000 days in the study of Lyszkowaska *et al* [28], patients were catheterized for a significantly longer duration in the remaining trials. Included trials also differed in the use of prophylactic antibiotics in the study population. Such inter-study heterogeneity was, however, expected as absolute similarities between RCTs conducted at different time points and different centers are not plausible. In our opinion, the included studies had sufficient similarities for the conduct of a meta-analysis, like analysis of the pediatric population with long-term central venous catheterization, use of similar concentration of taurolidine locking solution and more or less similar definition of outcomes.

The results of our study should be interpreted with the following limitations. Firstly, the strength of any meta-analysis depends upon the quality of the included studies. The results of our study should be interpreted with caution as the overall quality of studies was not high. The absence of adequate methods of randomization and the absence of blinding may have skewed the study results limiting the strength of the current evidence. Secondly, as discussed earlier, there was significant inter-study heterogeneity which may also have influenced outcomes. Thirdly, only four trials were available for analysis in our review. One of the studies included was underpowered [26]. Fourthly, the study population in the included studies was restricted to cancer and surgical patients. The efficacy of taurolidine lock solution in other patient populations is not completely known. Lastly, all included trials were conducted in European countries. There may be differences in CVC protocol in Europe vs other countries and the findings of this review cannot be generalized at this point.

Nevertheless, our review is the first meta-analysis on the efficacy of taurolidine lock solution in pediatric patients. Our results were synthesized only from RCTs to provide the highest level of evidence on the subject. The stability of our results on sensitivity analysis lends some credibility to our conclusions.

Within the limitations of our review, our study indicates that the use of taurolidine as a catheter locking solution may significantly reduce CRBSI in pediatric patients. Strong conclusions cannot be drawn due to the limited quality of the included studies. Further high-quality large scale RCTs are needed to corroborate our results.

## Supporting information

S1 Checklist (DOC)

S1 Table Search strategy of the review. (DOCX)
