# Supplementary material for: Taurolidine lock solution for catheter-related bloodstream infections in pediatric patients: A meta-analysis
Source: PLoS One. 2020 Apr 7;15(4):e0231110. doi: 10.1371/journal.pone.0231110 (PMC7138323; doi:10.1371/journal.pone.0231110)
Supplement: S1 Table — (DOCX) [file pone.0231110.s002.docx]

Supplementary material 1: Search strategy and results of PubMed database

| **Search number** | **Query** | **Search Details** | **PubMed Results** |
| --- | --- | --- | --- |
| **1** | (central venous catheters) AND (pediatrics) | (("central venous catheters"[MeSH Terms] OR (("central"[All Fields] AND "venous"[All Fields]) AND "catheters"[All Fields])) OR "central venous catheters"[All Fields]) AND (((("paediatrics"[All Fields] OR "pediatrics"[MeSH Terms]) OR "pediatrics"[All Fields]) OR "paediatric"[All Fields]) OR "pediatric"[All Fields]) | 2,201 |
| **2** | (central venous catheters) AND (children) | (("central venous catheters"[MeSH Terms] OR (("central"[All Fields] AND "venous"[All Fields]) AND "catheters"[All Fields])) OR "central venous catheters"[All Fields]) AND (((((("child"[MeSH Terms] OR "child"[All Fields]) OR "children"[All Fields]) OR "child s"[All Fields]) OR "children s"[All Fields]) OR "childrens"[All Fields]) OR "childs"[All Fields]) | 2,432 |
| **3** | (central venous catheters) AND (Catheter related infection) | (("central venous catheters"[MeSH Terms] OR (("central"[All Fields] AND "venous"[All Fields]) AND "catheters"[All Fields])) OR "central venous catheters"[All Fields]) AND (((("catheter-related infections"[MeSH Terms] OR ("catheter related"[All Fields] AND "infections"[All Fields])) OR "catheter related infections"[All Fields]) OR (("catheter"[All Fields] AND "related"[All Fields]) AND "infection"[All Fields])) OR "catheter related infection"[All Fields]) | 3,002 |
| **4** | (central venous catheters) AND (antimicrobial lock solution) | (("central venous catheters"[MeSH Terms] OR (("central"[All Fields] AND "venous"[All Fields]) AND "catheters"[All Fields])) OR "central venous catheters"[All Fields]) AND ((((((("anti infective agents"[Pharmacological Action] OR "anti-infective agents"[MeSH Terms]) OR ("anti infective"[All Fields] AND "agents"[All Fields])) OR "anti infective agents"[All Fields]) OR "antimicrobial"[All Fields]) OR "antimicrobials"[All Fields]) OR "antimicrobially"[All Fields]) AND "lock"[All Fields] AND (((((((((((("pharmaceutical solutions"[Pharmacological Action] OR "solutions"[MeSH Terms]) OR "solutions"[All Fields]) OR "solution"[All Fields]) OR "pharmaceutical solutions"[MeSH Terms]) OR ("pharmaceutical"[All Fields] AND "solutions"[All Fields])) OR "pharmaceutical solutions"[All Fields]) OR "solutal"[All Fields]) OR "solute"[All Fields]) OR "solute s"[All Fields]) OR "soluted"[All Fields]) OR "solutes"[All Fields]) OR "solution s"[All Fields])) | 132 |
| **5** | (central venous catheters) AND (antibiotic lock solution) | (("central venous catheters"[MeSH Terms] OR (("central"[All Fields] AND "venous"[All Fields]) AND "catheters"[All Fields])) OR "central venous catheters"[All Fields]) AND (((((((("anti bacterial agents"[Pharmacological Action] OR "anti-bacterial agents"[MeSH Terms]) OR ("anti bacterial"[All Fields] AND "agents"[All Fields])) OR "anti bacterial agents"[All Fields]) OR "antibiotic"[All Fields]) OR "antibiotics"[All Fields]) OR "antibiotic s"[All Fields]) OR "antibiotical"[All Fields]) AND "lock"[All Fields] AND (((((((((((("pharmaceutical solutions"[Pharmacological Action] OR "solutions"[MeSH Terms]) OR "solutions"[All Fields]) OR "solution"[All Fields]) OR "pharmaceutical solutions"[MeSH Terms]) OR ("pharmaceutical"[All Fields] AND "solutions"[All Fields])) OR "pharmaceutical solutions"[All Fields]) OR "solutal"[All Fields]) OR "solute"[All Fields]) OR "solute s"[All Fields]) OR "soluted"[All Fields]) OR "solutes"[All Fields]) OR "solution s"[All Fields])) | 98 |
| **6** | (central venous catheters) AND (Taurolidine citrate) | (("central venous catheters"[MeSH Terms] OR (("central"[All Fields] AND "venous"[All Fields]) AND "catheters"[All Fields])) OR "central venous catheters"[All Fields]) AND (("taurolidine"[Supplementary Concept] OR "taurolidine"[All Fields]) AND (((((("citrate s"[All Fields] OR "citrates"[MeSH Terms]) OR "citrates"[All Fields]) OR "citric acid"[MeSH Terms]) OR ("citric"[All Fields] AND "acid"[All Fields])) OR "citric acid"[All Fields]) OR "citrate"[All Fields])) | 27 |
| **7** | (central venous catheters) AND (Taurolidine) | (("central venous catheters"[MeSH Terms] OR (("central"[All Fields] AND "venous"[All Fields]) AND "catheters"[All Fields])) OR "central venous catheters"[All Fields]) AND ("taurolidine"[Supplementary Concept] OR "taurolidine"[All Fields]) | 45 |
| **8** | (central venous catheters) AND (Heparin) | (("central venous catheters"[MeSH Terms] OR (("central"[All Fields] AND "venous"[All Fields]) AND "catheters"[All Fields])) OR "central venous catheters"[All Fields]) AND ((((((((((((((("heparin"[MeSH Terms] OR "heparin"[All Fields]) OR "heparine"[All Fields]) OR "heparins"[All Fields]) OR "heparin s"[All Fields]) OR "heparinate"[All Fields]) OR "heparinated"[All Fields]) OR "heparines"[All Fields]) OR "heparinic"[All Fields]) OR "heparinisation"[All Fields]) OR "heparinised"[All Fields]) ) OR "heparinization"[All Fields]) OR "heparinize"[All Fields]) OR "heparinized"[All Fields]) OR "heparinizing"[All Fields]) | 695 |
| **9** | ((central venous catheters) AND (infection)) AND (prevention) | ((("central venous catheters"[MeSH Terms] OR (("central"[All Fields] AND "venous"[All Fields]) AND "catheters"[All Fields])) OR "central venous catheters"[All Fields]) AND ((((((((((((((((((((("infect"[All Fields] OR "infectability"[All Fields]) OR "infectable"[All Fields]) OR "infectant"[All Fields]) OR "infectants"[All Fields]) OR "infected"[All Fields]) OR "infecteds"[All Fields]) OR "infectibility"[All Fields]) OR "infectible"[All Fields]) OR "infecting"[All Fields]) OR "infection s"[All Fields]) OR "infections"[MeSH Terms]) OR "infections"[All Fields]) OR "infection"[All Fields]) OR "infective"[All Fields]) OR "infectiveness"[All Fields]) OR "infectives"[All Fields]) OR "infectivities"[All Fields]) OR "infects"[All Fields]) OR "pathogenicity"[MeSH Subheading]) OR "pathogenicity"[All Fields]) OR "infectivity"[All Fields])) AND ((((((((((((((((("prevent"[All Fields] OR "preventability"[All Fields]) OR "preventable"[All Fields]) OR "preventative"[All Fields]) OR "preventatively"[All Fields]) OR "preventatives"[All Fields]) OR "prevented"[All Fields]) OR "preventing"[All Fields]) OR "prevention and control"[MeSH Subheading]) OR ("prevention"[All Fields] AND "control"[All Fields])) OR "prevention and control"[All Fields]) OR "prevention"[All Fields]) OR "prevention s"[All Fields]) OR "preventions"[All Fields]) OR "preventive"[All Fields]) OR "preventively"[All Fields]) OR "preventives"[All Fields]) OR "prevents"[All Fields]) | 2,285 |
